# Granulomonocytapheresis for chronic inflammatory diseases and sepsis

**DOI:** 10.1186/s40560-025-00825-8

**Published:** 2025-10-03

**Authors:** Toshiaki Iba, Hideshi Okada, Takahiro Miki, Michio Mineshima, Ricard Ferrer

**Affiliations:** 1 Faculty of Medical Science, Juntendo University, 6-8-1 Hinode, Urayasu, Chiba 279-0013 Japan; 2 Department of Emergency and Disaster Medicine, Gifu University Graduate School of Medicine, Gifu, Japan; 3 Department of Clinical Engineer, Nihon University School of Medicine, 1-8-13 Kanda, Surugadai, Chiyoda-Ku, Tokyo, Japan; 4 Faculty of Medical Science, Juntendo University, 6-8-1 Hinode, Urayasu, Chiba 279-0013 Japan; 5 Intensive Care Department, Hospital Universitari Vall d’Hebron Universitat Autònoma de Barcelona, Barcelona, Spain

**Keywords:** Monocyte, Granulocyte, Hemofiltration, Ulcerative colitis, Cytokine

## Abstract

Granulomonocytapheresis (GMA) has long been used to treat refractory chronic inflammatory diseases. Recently, an exploratory clinical study showed that GMA was effective for sepsis, and its use has been approved in Japan. The purpose of this review is to spread the knowledge about GMA in chronic and acute inflammation. GMA is a selective extracorporeal therapy designed to remove activated granulocytes and monocytes, key drivers of inflammation in various immune-mediated diseases. Initially developed for ulcerative colitis, GMA has since demonstrated immunomodulatory effects in conditions such as Crohn’s disease, rheumatoid arthritis, and dermatologic disorders, by depleting activated myeloid cells and altering cytokine profiles, reducing tumor necrosis factor (TNF)-α, interleukin (IL)-6, and increasing IL-10. GMA aims to restore immune homeostasis without the systemic immunosuppression associated with pharmacologic agents. Recently, its application has expanded to critical care settings. In sepsis and cytokine storm syndromes, where overwhelming innate immune activation leads to organ dysfunction, GMA may offer therapeutic benefit. Preclinical models and pilot studies in septic patients suggest that GMA can reduce inflammatory mediators, improve hemodynamics, and support organ recovery. Reflecting this potential, GMA was approved for insurance reimbursement in Japan in August 2025 as adjunctive therapy for sepsis with systemic inflammation. Although GMA is a promising therapy for specific patients, there is limited supporting data, and its effect should be proven in future trials.

## Introduction

Chronic inflammatory diseases such as inflammatory bowel disease (IBD), rheumatoid arthritis (RA), and psoriasis represent a major clinical burden due to their persistent immune activation, relapsing course, and resistance to conventional therapies. These conditions are driven by complex interactions between innate and adaptive immune systems, with dysregulated responses of granulocytes and monocytes playing a central role in initiating and perpetuating inflammation. Although pharmacological treatments, including corticosteroids, immunosuppressants, and biologic agents targeting specific cytokines, have significantly improved disease control, a substantial proportion of patients remain refractory, intolerant, or at risk of adverse effects such as infection, malignancy, or systemic immunosuppression.

In this context, nonpharmacological interventions that modulate immune activation while minimizing systemic toxicity have emerged as attractive therapeutic options. Granulomonocytapheresis (GMA) is a selective apheresis technique developed to remove activated granulocytes and monocytes from the peripheral circulation. Utilizing an extracorporeal column containing cellulose acetate beads (Adacolumn^®^, JIMRO Co., Ltd., Takasaki, Japan), GMA selectively adsorbs these myeloid-lineage cells through interactions with complement and Fcγ receptors. Unlike plasma exchange or other non-selective apheresis modalities, GMA preserves lymphocytes and humoral immunity, thereby offering a targeted yet immune-preserving approach.

Initially introduced in Japan for the treatment of ulcerative colitis (UC) in the late 1990s [1, 2], GMA has since been applied to other immune-mediated disorders such as Crohn’s disease, rheumatoid arthritis, and dermatologic conditions, including psoriasis and pyoderma gangrenosum. Clinical studies have demonstrated its efficacy in inducing remission, reducing corticosteroid dependence, and modulating systemic cytokine profiles, with a favorable safety record and low risk of opportunistic infections. These outcomes have led to its approval and insurance coverage in Japan and selected countries in Europe for specific indications [3].

Importantly, the immunomodulatory potential of GMA has attracted growing interest in critical care and infectious disease settings, particularly in conditions characterized by hyperinflammation and cytokine storms. In recent years, exploratory studies have investigated its application in severe COVID-19 and septic shock [4, 5]. These studies suggest that early depletion of activated myeloid cells may mitigate tissue injury, reduce cytokine levels, and improve organ function [6]. Based on this accumulating evidence and expert consensus, in August 2025, the Japanese Ministry of Health, Labour, and Welfare (MHLW) officially approved granulomonocytapheresis for insurance reimbursement as an adjunctive treatment in patients with sepsis and systemic inflammation [7]. This regulatory milestone highlights a paradigm shift in the therapeutic landscape, recognizing extracorporeal immune modulation as a viable strategy in managing systemic inflammatory disorders beyond autoimmunity [8].

Despite its potential and feasibility, GMA remains underutilized globally due to cost considerations, limited familiarity outside of East Asia, and a lack of large-scale randomized trials in many indications [9]. Moreover, its exact positioning relative to emerging biologics and small-molecule inhibitors remains to be defined. Nevertheless, GMA offers unique advantages, non-pharmacological, low immunosuppressive burden, and repeatable therapy, which align well with personalized medicine approaches, particularly in refractory or high-risk populations [10].

This review summarizes the current understanding of GMA, including its mechanisms, preclinical and clinical data, and expanding therapeutic scope, with a particular focus on its recent application to sepsis. We aim to provide clinicians and researchers with an updated overview of this evolving modality and its potential integration into multidisciplinary inflammatory disease management.

## Background and mechanism of action

### Principles of GMA

**Fig. 1:**
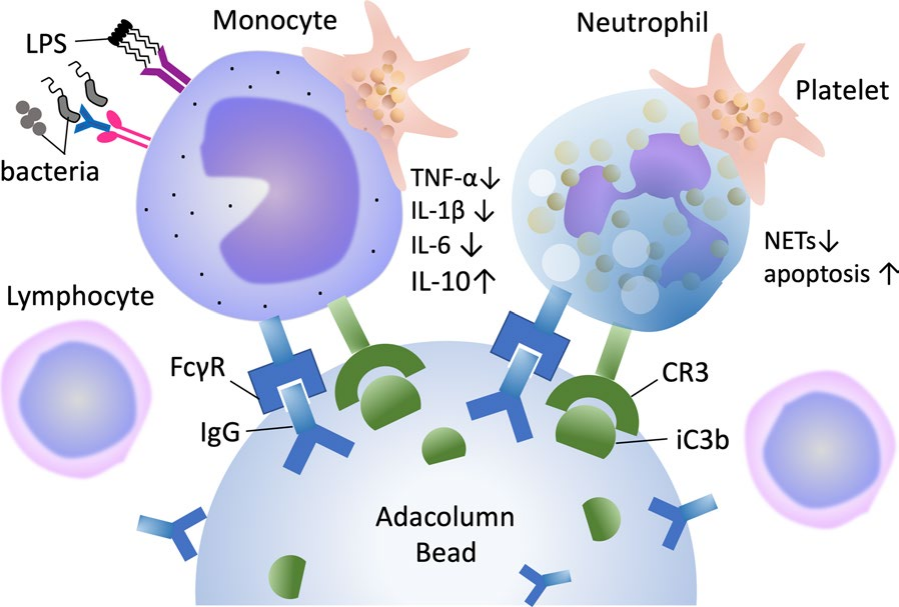
Mechanism of granulomonocytapheresis. Mechanism of action of Adacolumn hemoadsorption. Cellulose acetate beads within the column become coated with immunoglobulin G (IgG) and complement fragment iC3b, creating an opsonized surface. Activated granulocytes (neutrophils, eosinophils) and monocytes adhere via Fcγ receptors (FcγR) and complement receptor 3 (CR3), leading to their selective removal from circulation. This process reduces pro-inflammatory cytokines (TNF-α, IL-1β, IL-6), neutrophil extracellular traps (NETs), and bacterial lipopolysaccharide (LPS) activity, while increasing anti-inflammatory cytokine IL-10 and promoting apoptosis of activated cells. Regulatory immune modulation also affects lymphocyte populations

GMA achieves targeted leukocyte depletion by selectively removing activated granulocytes and monocytes from the peripheral blood using cellulose acetate beads within an extracorporeal column. These beads bind leukocytes via complement (C3b) and Fcγ receptors, enabling the physical adsorption of pro-inflammatory cells while sparing lymphocytes [11, 12] (Fig. 1). The removed cells are known to express high levels of CD11b and release cytokines such as TNF-α and IL-1β. Depletion of these cell populations reduces circulating cytokine levels and shifts the immune response toward resolution of inflammation without causing global immunosuppression [13]. This cell-selective approach underpins the therapeutic rationale of GMA.

### Targeted leukocytes

An extracorporeal apheresis device is specifically engineered to selectively deplete activated myeloid-lineage leukocytes from the peripheral blood, particularly neutrophils and monocytes. These two cell populations are essential players in the innate immune response and are critically involved in the pathogenesis of many inflammatory diseases. When activated, neutrophils and monocytes express high levels of surface molecules such as CD11b/CD18 (Mac-1), Fcγ receptors, and complement receptors, which facilitate their interaction with the cellulose acetate beads. These beads are designed to mimic immune complex-coated surfaces and bind circulating leukocytes via complement-opsonized and Fc receptor-mediated mechanisms.

Approximately 60–70% of the leukocytes adsorbed during a single session of GMA are neutrophils [12]. These cells are major producers of pro-inflammatory cytokines such as tumor necrosis factor-alpha (TNF-α), interleukin (IL)-1β, and IL-8, as well as tissue-damaging enzymes and reactive oxygen species [14, 15].

Monocytes, which constitute roughly 20–30% of the adsorbed cells, also contribute to systemic inflammation through cytokine production and their capacity to differentiate into macrophages and dendritic cells [2, 12]. Importantly, the Adacolumn^®^ preferentially binds activated rather than resting leukocytes, enhancing its selectivity and reducing off-target effects [11, 16].

Lymphocytes and platelets, in contrast, are largely spared during GMA because they lack significant expression of FcγR and do not readily interact with the beads [12]. As a result of lymphocyte preservation, the adaptive immune system remains intact, which helps minimize the risk of broad immunosuppression, a key advantage over conventional pharmacologic therapies. This targeted removal of inflammatory cells while preserving overall immune competence underlies the therapeutic appeal of GMA in both chronic autoimmune diseases and acute inflammatory conditions such as sepsis [17]. As for platelets, a sepsis study by Nishida et al. [5] reported the decreased platelet count from 118.5 × 10^3^/μL at baseline to 58.5 × 10^3^/μL on Day 3. Although the mechanism is unclear, the interaction between activated leukocyte–platelet may relate to the platelet elimination.

### Immunomodulatory effects

GMA exerts immunomodulatory effects by selectively removing activated neutrophils and monocytes, which are major sources of pro-inflammatory cytokines such as TNF-α, IL-1β, and IL-6 [15]. This depletion leads to a reduction in systemic cytokine levels and an associated decrease in inflammation. In addition, GMA promotes a shift toward anti-inflammatory immune profiles, including increased levels of IL-10 and regulatory T cell activity [18]. The therapy also induces apoptosis in circulating activated leukocytes and promotes the release of immature, less reactive myeloid cells from the bone marrow [19]. These effects contribute to immune homeostasis without the generalized immunosuppression seen with pharmacologic therapies.

## Preclinical studies

Although most data on GMA derive from clinical studies, several animal models have provided insight into its biological effects.

### Colitis models

In dextran sulfate sodium (DSS)-induced colitis in mice, the transfusion of leukocytes pre-treated with cellulose acetate beads resulted in amelioration of colitis symptoms. Inflammatory cytokines such as TNF-αwas significantly decreased, while histological inflammation scores improved [20].

### Arthritis models

Kyogoku et al. [21] reported the selective removal of activated immune cells by GMA in the rat model of rheumatoid arthritis. The column depleted neutrophils, monocytes, natural killer (NK) cells, and B cells, but not T cells. These results indicated beyond cell removal, GMA modifies blood components, potentially restoring immune balance and promoting arthritis healing. Nakazato et al. [22] have demonstrated that Adacolumn® adsorbs about 20% of circulating granulocytes and monocytes, significantly reducing their migration to inflamed sites in arthritic rats, which may explain its rapid anti-inflammatory effects in rheumatoid arthritis. They also showed that this hemoadsorption does not affect T cell migration but markedly suppresses their antigen reactivity.

### Mechanistic insights

Ex vivo studies in human blood and animal models revealed that cellulose acetate beads stimulate monocytes to release IL-10 and inhibit their differentiation into pro-inflammatory macrophages [23]. Additionally, the adsorptive surface reduced neutrophil extracellular trap (NET) formation and mitigated oxidative burst activity [19]. These findings support the hypothesis that GMA acts not only by physical depletion but also through dynamic immune reprogramming.

## Effects on inflammatory bowel diseases and other inflammatory diseases

Clinical research on GMA has been most extensively conducted in patients with UC, where it has demonstrated both safety and efficacy [24–26]. Early multicenter studies in Japan reported high remission rates and steroid-sparing effects in patients with moderate to severe UC who were either steroid-dependent or steroid-refractory [27]. A pivotal randomized, double-blind, sham-controlled trial by Hanai et al. [28] showed that GMA significantly improved clinical response rates compared to prednisolone therapy, with notable reductions in disease activity scores and mucosal inflammation. Importantly, the therapy was well tolerated, with a favorable safety profile and low incidence of adverse events.

Further clinical studies extended GMA’s application to special populations, including pediatric patients and those with extraintestinal manifestations [29]. GMA has also been explored in Crohn’s disease and demonstrated potential benefits in select cases [30], although outcomes have been more variable than in UC [31]. In rheumatoid arthritis, patients significantly altered serum cytokines, reducing TNF-α, IL-15, and RANTES (Regulated on activation, normal T expressed and secreted), while increasing IL-10, which correlated with clinical improvement, especially in treatment responders [32]. Another trial combining GMA with disease-modifying anti-rheumatic drugs (DMARDs) has shown additive effects on symptom relief and inflammatory markers, especially in patients resistant to biologics [29].

Meta-analyses have further confirmed the efficacy of GMA in inducing remission and maintaining clinical response in UC [33]. However, the benefits appear more pronounced in steroid-naïve or moderately active disease, and less consistent in patients with extensive colonic involvement or high inflammatory burden. The optimal treatment regimen (e.g., number and frequency of sessions) remains under investigation, with evidence suggesting that intensive protocols may be more effective in refractory cases.

Collectively, clinical studies highlight GMA as a safe and effective adjunctive therapy in various inflammatory diseases, with particular promise in UC and emerging roles in systemic inflammatory syndromes [2, 9, 34] (Table 1). However, large-scale randomized controlled trials, particularly in critical care populations, are needed to further validate its efficacy and define its place in modern treatment algorithms.

**Table 1:** Effects of granulomonocytapheresis on ulcerative colitis and Crohn’s disease

Study/Source	Patient Population	Comparator/Setting	Effectiveness (Remission/Response)	Additional Notes/Safety	Citations
Fukuda et al., Open-label, prospective 2004	Refractory Crohn’s disease (CD), multicenter	Open-label, prospective	Clinical response: 57% (35/61), remission: 41% (25/61); significant decrease in CDAI and CRP after 5 sessions	Well tolerated, no severe adverse events	[24]
Yamamoto et al., 2018	Moderate-severe UC, multicenter	Retrospective, patient factors	Clinical response at end of GMA: 46.6% (69/148) overall; predictors: younger age, lower disease activity, first episode	Few adverse events; responder profile explored	[25]
Sakuraba et al., 2009	Active ulcerative colitis	Intensive (2 × /wk) vs routine (1 × /wk) GMA	Remission at 2 weeks: intensive 71.4% (25/35) vs weekly 17.6% (6/34), *P* < 0.001; faster symptom improvement	Intensive GMA more effective, similarly safe	[26]
Fukunaga et al., 2012 (RCT, double blind, sham-controlled)	Ulcerative colitis (maintenance therapy)	Sham apheresis	Maintenance of remission significantly higher with GMA vs sham at 12 months	Prospective, randomized; good safety profile	[27]
Hanai et al., 2004	Corticosteroid-dependent, moderately severe UC	Prednisolone	Comparable remission rates; fewer side effects with GMA compared to prednisolone	GMA as steroid-sparing therapy	[28]
Hidaka et al., 2001	Rheumatoid arthritis	Filtration leukocytapheresis	Significant decrease in pro-inflammatory cytokines (e.g., IL-1, TNF-alpha) in serum and synovial fluid	Evidence for cytokine modulation; safe procedure	[32]
Liu et al., 2016 (Meta-analysis)	Inflammatory bowel disease (IBD)	Various comparators	Confirmed efficacy and safety of selective GMA for inducing remission in IBD	Meta-analysis supports favorable risk–benefit	[34]
Yoshino et al. Meta-analysis, 2014	Ulcerative colitis	Corticosteroids	OR: 2.23 (95% CI 1.38–3.60) for remission; intensive apheresis (≥ 2/week) more effective (OR: 2.10)	Lower adverse events than steroids	[9]
Kiss et al. Meta-analysis, 2021	Ulcerative colitis	Conventional therapy	OR: 1.93 (1.28–2.91) for induction; OR: 8.34 (2.64–26.32) for maintenance	No significant adverse event increase	[33]
Sands et al. RCT, 2008	Moderate-severe ulcerative colitis	Sham apheresis	Remission: 17% vs 11% (*P* = 0.361) not significant; response: 44% vs 39% (*P* = 0.62)	Well tolerated, no efficacy difference	[35]
Imperiali et al. multicenter, 2017	Steroid-dependent, azathioprine-intolerant UC	–	Steroid-free remission at 12 months: 36%	Alternative to biologics or surgery	[36]
Sands et al. RCT, 2013	Moderate-severe Crohn’s disease (CDAI 220–450)	Sham apheresis	Remission: 17.8% GMA vs 19.2% sham (ns); response: 28% GMA vs 26.9% sham	No difference in adverse events	[30]
Yoshimura et al. open-label prospective multicenter study, 2015	Crohn’s disease, active	Intensive vs routine GMA	Clinical remission higher in 2/week group; well tolerated	GMA effective and safe in active Crohn’s disease	[31]
Ueno et al. 2024, multicenter retrospective pilot study	Crohn’s disease, refractory, loss of response	Biologics + GMA	Remission 25%, response 68.8%; biologics maintained in 36.4% after GMA in previous LOR cases	Safe, suggests benefit as adjunct	[37]

*OR* odds ratio, *CI* Confidence Interval, *CDAI* Crohn’s disease activity index, *GMA* granulomonocytapheresis, *LOA* limits of agreement

GMA exerts notable extraintestinal effects beyond the gut, particularly by modulating systemic immune responses. It reduces circulating activated granulocytes and monocytes, leading to decreased pro-inflammatory cytokines such as TNF-α and IL-1, as demonstrated in rheumatoid arthritis, psoriasis, and pyoderma gangrenosum. Clinical studies show GMA improves symptoms in refractory autoimmune and neutrophil-driven conditions with favorable safety, often sparing systemic immunosuppressants (Table 2). These immunomodulatory effects promote resolution of skin lesions, joint inflammation, and ulcer healing, highlighting GMA as a promising adjunct or alternative therapy for extraintestinal immune-mediated manifestations associated with inflammatory diseases.

**Table 2:** Effects of granulomonocytapheresis on rheumatoid arthritis, psoriasis, pyoderma gangrenosum

Authors	Patient Population	Setting	Effectiveness (Remission/Response)	Additional Notes/Safety	Citation
Sanmartí et al	Rheumatoid arthritis, refractory to DMARDs	Open-label, no direct comparator, multicenter	ACR20 achieved in 40.7% (intention to treat), 50% for completers; EULAR response in 44.4%; also effective after biologics fail	Well tolerated; 1 serious event (catheter-related sepsis)	[38]
Cuadrado et al	Rheumatoid arthritis, IBD (Review)	–	GMA is useful for refractory RA, improves clinical activity in some patients	Generally well-tolerated; used for drug-sparing effect	[39]
Kashiwagi et al	Immune arthritis model (rabbit)	–	Showed anti-inflammatory effect, reduced infiltration and cytokines	Experimental model	[40]
Seishima et al	Psoriatic arthritis, refractory to drugs	Case series	Effective for plaque-type skin eruptions in all, joint symptoms in 3/4 (mild only)	Effective for skin, limited effect on severe joint	[41]
Kanekura et al	4 psoriatic arthritis patients	Case series	remarkable clearing of joint pain	Effective for skin	[42]
Sakanoue et al	GPP, lost response to biologics	Case series	GMA “regained” response in 2 refractory GPP patients on biologics; rapid lesion improvement	Safe for elderly, children, pregnant, combination use	[43]
Higashi et al	Pyoderma gangrenosum	Prospective single-center	Complete response in 8, near complete in 3, partial in 2 of 15; some stable, 2 progressed	No severe adverse events, safe	[44]
Seishima et al	Pyoderma gangrenosum + UC/RA, refractory	Case series	Skin lesion reduction and re-epithelialization in all 3 cases after 10–11 sessions	No adverse events over 8 months	[45]

*GMA* granulomonocytapheresis, *DMARDs* disease-modifying antirheumatic drugs,

*ACR* American College of Rheumatology criteria, *EULAR* European League Against Rheumatism, *IBD* inflammatory bowel disease, *RA* rheumatoid arthritis, *GPP* Generalized pustular psoriasis, *UC* ulcerative colitis

## Effects on sepsis

Sepsis is a life-threatening condition characterized by dysregulated systemic inflammation resulting from infection, leading to organ dysfunction and high mortality. Despite advances in antimicrobial therapy and supportive care, immunomodulatory strategies for sepsis remain a major unmet need [46]. Recently, GMA was proposed as a therapeutic approach to modulate the hyperinflammatory response characteristic of early sepsis.

### Pathophysiological rationale

Activated neutrophils and monocytes are central to the pathogenesis of sepsis-induced tissue damage. Their overactivation leads to excessive release of cytokines (e.g., TNF-α, IL-1β, IL-6), proteases, and reactive oxygen species, which contribute to endothelial injury, coagulopathy, and organ dysfunction [47]. GMA's capacity to selectively deplete these cells without broadly suppressing adaptive immunity offers a mechanistically targeted intervention. Additionally, GMA may shift monocyte polarization toward anti-inflammatory phenotypes and reduce circulating DAMPs and cytokine storm mediators [48].

### Preclinical evidence

Hara et al. [49] developed a novel immunomodulating blood purification system combining GMA using Adacolumn® with a cytokine-adsorbing hemofilter (AN69ST membrane). Using fresh porcine blood stimulated by lipopolysaccharide (LPS), the system selectively removed activated granulocytes and monocytes, reducing their phagocytic activity and adhesiveness while sparing lymphocytes. They claimed that this system has a favorable immunomodulatory effect by suppressing the excessive increase of proinflammatory cytokines, which leads to the avoidance of excessive inflammation and organ dysfunction. Another experimental porcine model of septic shock induced by peritonitis, GMA was shown to attenuate the systemic inflammatory response. Specifically, GMA-treated animals exhibited reduced plasma levels of TNF-α and IL-6, preserved hemodynamics, and attenuated lung injury compared to controls. Histological analyses demonstrated decreased leukocyte infiltration in lung and kidney tissues [50]. Another rodent study demonstrated that early GMA for 2 h at 18 h after cecal ligation and puncture. The treatment significantly reduced serum cytokine levels and improved survival [6].

### Clinical observations

Clinical data on GMA in sepsis are still limited and primarily observational. In a small case series of septic patients with multiple organ failure who received GMA as adjunctive therapy, reductions in vasopressor requirements, serum cytokines, and procalcitonin levels were reported. Patients also showed improvements in SOFA scores (the change of SOFA score from baseline to Day 7 was − 5 (*P* < 0.01) and lactate clearance, suggesting enhanced hemodynamic stability and organ perfusion [5, 8]. In this study, GMA was generally set at a blood flow rate of 50 mL/min for 120 min, using five columns within 3 days. The second round commenced 12 h (± 6 h) after the first round, the third round commenced 24 h (± 6 h) after the first round, and the fourth and fifth rounds commenced 24 h (± 6 h) after the previous use.

During the COVID-19 pandemic, GMA was explored as a treatment for patients with severe SARS-CoV-2 pneumonia exhibiting hyperinflammatory features similar to bacterial sepsis. A pilot study showed that GMA removed granulocyte subsets expressed CD11b, CD16, and CD66b, and weakly expressed CD11c, consistent with mature and activated neutrophils, and monocyte subsets strongly expressed CD14, suggesting that GMA can modulate the hypercytokinemia in COVID-19 [51]. In a similar manner, GMA is expected to be effective for the treatment of multisystem inflammatory syndrome in adults (MIS-A) following COVID-19 [4].

### Current limitations and future potential

Despite promising preliminary data, robust randomized controlled trials are lacking. Key limitations include logistical challenges of apheresis in critically ill patients, timing of intervention, and patient selection. Future studies should focus on early-phase sepsis, using biomarkers such as elevated neutrophil counts or cytokine panels to identify responders. In particular, integration with existing sepsis bundles and antimicrobial therapy should be carefully evaluated. Overall, GMA represents a promising adjunctive strategy for immunomodulation in early sepsis. Its selective targeting of innate immune overactivation may complement current sepsis management, but well-designed clinical trials are essential to define its role [52].

## Future perspectives

### Biomarker-guided therapy

Identifying biomarkers predictive of GMA response is critical. Elevated peripheral neutrophil counts, fecal calprotectin, and circulating cytokine profiles may help stratify patients who would benefit most [53]. Transcriptomic analysis of leukocyte subtypes could guide personalization of therapy [54].

### Safety features and health economics

In the clinical studies performed in Japan, adverse events of GMA are reported to be 12.2% and none are serious [5]. However, the risk of worsening the infection and thrombocytopenia should be carefully examined in future studies.

Regarding cost, GMA is costlier than conventional therapies and is not universally reimbursed. In the cases of UC, cost-effectiveness analyses factoring in reduced hospitalizations and steroid use are needed to justify broader implementation. Training and infrastructure development are essential in low-resource settings [55].

## Comparing with other columns

Comparing GMA with other treatments, such as PMX-HP and AN69ST. These extracorporeal devices also exert cytapheretic or immunomodulatory effects. Polymyxin B-immobilized fiber column (PMX-HP) is primarily designed for endotoxin removal, but it also adsorbs activated monocytes and neutrophils, thereby exerting indirect immunomodulation. The recent success in the TIGRIS trial is an epoch-making event that sheds light on hemoperfusion for sepsis [56]. The AN69ST membrane, developed for cytokine adsorption, has also been reported to capture activated leukocytes and platelets [57]. Although these systems share mechanistic overlap with GMA, Adacolumn is unique in its selective depletion of activated granulocytes and monocytes, with the goal of rebalancing immune homeostasis rather than broad endotoxin or cytokine removal. We added the above description to the text.

## Conclusion

GMA represents a promising non-pharmacologic option for managing refractory inflammatory diseases. Its unique mechanism of selectively depleting activated myeloid cells while preserving immune competence offers a distinct therapeutic advantage. Clinical studies in UC and rheumatoid arthritis confirm its efficacy and safety, though responses vary. Limited evidence in sepsis is encouraging but insufficient for routine use. Moving forward, biomarker-driven patient selection, combination therapy strategies, and cost-effective deployment will be critical to integrating GMA into mainstream clinical practice.

## Data Availability

Not applicable.
